# Hyperoxia Exposure Impairs Nephrogenesis in the Neonatal Rat: Role of HIF-1α

**DOI:** 10.1371/journal.pone.0082421

**Published:** 2013-12-17

**Authors:** Constantin R. Popescu, Megan R. Sutherland, Anik Cloutier, Geneviève Benoît, Mariane Bertagnolli, Catherine Yzydorczyk, Nathalie Germain, Véronique Phan, Martine Lelièvre-Pegorier, Hervé Sartelet, Anne Monique Nuyt

**Affiliations:** 1 Sainte-Justine University Hospital and Research Center, and the Department of Pediatrics, Université de Montréal, Montreal, Quebec, Canada; 2 Sainte-Justine University Hospital and Research Center, and the Department of Pathology, Université de Montréal, Montreal, Quebec, Canada; 3 INSERM U872, Centre de Recherche des Cordeliers, Université Pierre et Marie Curie – Paris 6, and Université Paris Descartes UMR S 872, Paris, France; The University of Manchester, United Kingdom

## Abstract

Preterm neonates are exposed at birth to high oxygen concentrations relative to the intrauterine environment. We have previously shown in a rat model that a hyperoxic insult results in a reduced nephron number in adulthood. Therefore, the aim of this study was to determine the effects of transient neonatal hyperoxia exposure on nephrogenesis. Sprague-Dawley rat pups were raised in 80% O_2_ or room air from P3 to P10. Pups (n = 12/group, 6 males and 6 females) were sacrificed at P5 (during active nephrogenesis) and at P10 (after the completion of nephrogenesis). Hyperoxia exposure resulted in a significant reduction in both nephrogenic zone width and glomerular diameter at P5, and a significantly increased apoptotic cell count; however, nephron number at P10 was not affected. HIF-1α expression in the developing kidney was significantly reduced following hyperoxia exposure. Systemic administration of the HIF-1α stabilizer dimethyloxalylglycine (DMOG) resulted in enhanced expression of HIF-1α and improved nephrogenesis: kidneys from hyperoxia-exposed pups treated with DMOG exhibited a nephrogenic zone width and glomerular diameter similar to room-air controls. These findings demonstrate that neonatal hyperoxia exposure results in impaired nephrogenesis, which may be at least in part HIF-1α-mediated. Although nephron number was not significantly reduced at the completion of nephrogenesis, early indicators of maldevelopment suggest the potential for accelerated nephron loss in adulthood. Overall, this study supports the premise that prematurely born neonates exposed to high oxygen levels after birth are vulnerable to impaired renal development.

## Introduction

Preterm neonates are often born at a time when kidney development is still ongoing, as nephrogenesis (the development of nephrons) is not normally completed until 34-36 weeks gestation [1], [2]. Development of the kidneys continues after birth in preterm neonates [2]; however, glomerular abnormalities and reduced glomerular formation have been observed in this population indicating that postnatal nephrogenesis is probably impaired [2], [3]. Importantly, hypertension [4]–[6], reduced kidney size [7]–[9] and impaired renal function [10], [11] have been observed in children and adults that were born preterm, highlighting the long-term consequences of preterm birth on renal health.

The cause of impaired renal development following preterm birth, and the mechanisms by which this may program for adult renal disease are largely unknown; exposure to oxygen (O_2_) in the extrauterine environment, however, is likely to be a contributing factor [12]–[14]. Infants are exposed upon birth to supraphysiological concentrations of O_2_ compared to intrauterine life [15]. This results in oxidative stress of the newborn [16], [17], with preterm neonates particularly susceptible due to their low antioxidant levels [18], [19]. Oxidative stress has been implicated in a number of common diseases of prematurity including retinopathy of prematurity, bronchopulmonary dysplasia, and necrotising enterocolitis [20], [21].

Importantly, we have previously shown that early life exposure to hyperoxia in rats (80% O_2_ from postnatal day 3–10, a time when nephrogenesis is still ongoing) led to hypertension, and a 25% reduction in nephron number in adulthood [13]; underlying these findings may be a disruption to nephrogenesis. In this regard, rat metanephric organ culture studies have shown that both vasculogenesis and tubulogenesis are enhanced when tissues are kept under low (1–3%), rather than standard (21%), O_2_ concentrations [22]. Cellular oxygen homeostasis is predominantly controlled by the hypoxia inducible factor-1α (HIF-1α) transcription factor, with high oxygen levels resulting in the degradation of HIF-1α protein via prolyl-4-hydroxylase (PHD)-dependent interactions with the von Hippel-Lindau (pVHL) ubiquitin E3 ligase complex [23], [24]. HIF-1α is known to be essential for organogenesis [25], [26], by regulating the expression of numerous factors involved in angiogenesis, cellular proliferation, and apoptosis [27], [28]; however, to date no direct link between hyperoxia and impaired nephrogenesis has been established.

The aim of the current study, therefore, was to characterise the impact of transient hyperoxia exposure (80% O_2_ from P3 to P10) on nephrogenesis in the neonatal rat. Considering that HIF-1α controls the expression of a number of key factors involved in organogenesis, we further aimed to examine the role of the transcription factor on nephrogenesis during hyperoxia exposure. As HIF-1α is post-transcriptionally regulated by PHD, we assessed the effect of systemic administration of a PHD inhibitor, dimethyloxalylglycine (DMOG), in order to test the hypothesis that counteracting the oxygen-induced downregulation of HIF-1α activity would prevent any adverse effects of hyperoxia on nephrogenesis in this model.

## Methods

### Animals

All studies were approved by the Animal Care Committee of the CHU Sainte-Justine, and the treatment and care of the animals was in accordance with the principles of the *Guide for the Care and Use of Experimental Animals* from the Canadian Council on Animal Care. Sprague-Dawley rat pups (Charles River; St.-Constant, Québec, Canada) were born naturally at term and maintained in 80% O_2_ (mixture of medical grade 100% O_2_ and room air; Oxycycler ProOx model 110, Biosherix, Lacona, NY, USA), or room air, from the 3^rd^ (P3) to the 10^th^ (P10) day of life [13].

### Experimental groups

Hyperoxia-exposed pups were continuously exposed to 80% O_2_ from P3 to P10 (O_2_-exposed group (H); n  =  3 litters). To avoid severe O_2_ toxicity, the mother of the O_2_-exposed litter was interchanged every 12 hours with another dam in room air. The pups of the dam used for interchange served as a control for the effects of the dam being exposed to hyperoxia, and also the stress of the dam interchange, but themselves were maintained in room air (normoxia-hyperoxia group (NH); n  =  3 litters). Normal control litters were kept with their dams in room air (normoxia not interchanged group (NNI); n  =  3 litters). All litters were equalised to n = 12 at P3, and there was no difference in the number of males/females (5-7 of each sex per litter) or in the survival of the pups between groups. In all groups, 2 male and 2 female pups, selected at random, were analysed at P5 (n = 12/group); the remainder of pups were grown until P10, with 1–2 male and female pups selected for analysis (n = 12/group). Pups were sacrificed and kidneys collected for analysis at P5 and P10, with body and kidney weights recorded at the two time points. For all kidney analyses, researchers were blinded to the experimental group assignment of the animals.

In a separate series of experiments, 1–2 male and 1–2 female pups were selected at random from H and NH litters (n = 4 litters/group) and administered either the prolyl hydroxylase (PHD) inhibitor dimethyloxalylglycine (DMOG; Cayman Chemical Company; Ann Arbor, MI, USA) at a dosage of 200 μg/g of body weight [29] (H+DMOG and NH+DMOG treatment groups; n  =  6 males and 6 females per group), or an equivalent volume of saline solution (H and NH control groups; n  =  6 males and 6 females per group). Pups received the DMOG or saline via intraperitoneal injection at P2 (24 hours before hyperoxic exposure) and P4 (after the first 24 hours of hyperoxic exposure). Pups were sacrificed and kidneys collected for analysis at P5; the remainder of pups were grown to P10 (with 1 male and 1 female pup per litter selected for analysis, n = 12/group). During all kidney analyses, researchers were blinded as to the experimental grouping of the animals.

### Histomorphometry

Kidneys were harvested from P5 and P10 pups, weighed, fixed in formaldehyde and paraffin-embedded whole. 4μm sections from the central region of the kidney (across the full coronal plane) were haematoxylin phloxine saffron-stained and examined by optical microscopy. For each section, three microphotographs from the anterior, posterior, and mediolateral regions of the kidney (each including the full thickness of the cortex) were obtained with a digital camera at a magnification of 100X (AxioCam; Zeiss, Germany). ***Width of the renal cortex and nephrogenic zone:*** Using image analysis software (Image Pro Plus v. 6.3 for Windows), the width of the renal cortex (from the cortico-medullary junction to the superficial edge of the outer renal cortex) in pups at P10, and the width of the nephrogenic zone (area of growth in the outer renal cortex) in pups at P5 were determined. Five measurements of each parameter were recorded in each of the three fields of view (15 measurements per kidney) and then averaged to determine the mean width per kidney. ***Glomerular diameter:*** In each of the three fields of view examined, 5 mature glomeruli (15 per kidney) were selected at random for analysis of glomerular diameter. Using image analysis software (Image Pro Plus v. 6.3 for Windows) the diameter of each glomerulus in cross-section was measured, then averaged to determine the mean glomerular diameter per kidney.

### Glomerular number and density

The right kidney from saline/DMOG treated NH and H animals at P10 (a time point after the completion of nephrogenesis) were paraffin-embedded whole, and sectioned exhaustively at 7 µm. Every 20^th^ section was collected and stained with haematoxylin and eosin, imaged in parts then merged using Adobe Photoshop (CS6 Extended, v. 13.0.1). The Photoshop count tool was utilised to mark and count each individual glomerulus, then the result for all serial sections were combined (indicating the number of glomeruli counted in 5% of the kidney). Sections were 140µm apart to avoid counting any glomeruli more than once. To estimate the total number of nephrons per kidney, the formula by Murawski *et al.* [30] was employed: N_glom_  =  f × 0.4 × NN where f is inverse of the fraction of the kidney sampled, 0.4 is a constant, and NN is the number of glomeruli counted. Estimated total nephron number was then divided by kidney weight to determine the density of glomeruli in the kidney.

### Assessment of apoptosis

The extent of apoptosis was assessed using TUNEL assays on 4µm deparaffinised sections of kidney, from animals at P5. Sections were stained using the *In Situ* Cell Death Detection POD Kit (Roche Diagnostics; Laval, Québec, Canada) according to the manufacturer’s instructions, and counterstained with methyl green. For each section, three microphotographs of the nephrogenic zone were obtained from from the anterior, posterior, and mediolateral regions of the kidney (400X magnification). A circular boundary was superimposed on each image, creating a field of view with a diameter of 200 µm that was inclusive of 3 developing glomeruli (vesicle, comma-shaped or S-shaped bodies). For each photograph, all apoptotic cells (positive for TUNEL staining) within the field of view were counted, and the number of apoptotic cells per region of nephrogenic zone was then averaged per kidney. The number of apoptotic cells only present within the developing glomeruli was also assessed.

### Assessment of HIF-1α expression

**Immunohistochemisty.** Immunohistochemical localization of HIF-1α was performed on 4µm deparaffinised sections of kidney from animals at P5. Using the Ultraview Universal DAB detection kit (Ventana Medical Systems, Tuscon, AR, USA), mouse monoclonal anti-human HIF-1α (α67 at 1∶10 000 dilution; Novus Biologicals, Littleton, CO, USA) was applied for 32 min, followed by application of Ultraview Universal DAB detection kit according to the manufacturer’s instructions. Normal rabbit IgG (1∶10 000 dilution) served as a negative control. Two investigators, each blinded to the experimental groups, independently evaluated immunostaining scores for HIF-1α in the nephrogenic area and the cortical interstitium by a semi-quantitative optical assessment of the percentage of positive cells in each tissue section: 0  =  all cells negative, 1+  =  < 25% of positive cells, 2+  =  25 to 50% of positive cells, 3+  =  50 to 75% of positive cells and 4+  =  > 75% of positive cells. **Western blot.** Kidneys from P5 saline/DMOG treated NH and H animals (6 males and 6 females per group) were homogenized in nuclear extract buffers (Buffer A: 10 mM HEPES, 1.5 mM MgCl_2_, 10 mM KCl, 0.5 mM DTT and 10% Igepal; Buffer B: 5 mM HEPES, 1.5 mM MgCl_2_, 300 mM NaCl, 0.2 mM EDTA, 0.5 mM DTT and 26% glycerol), complemented with proteases inhibitors and 0.5 mM PMSF (as described by Li *et al*. [31]). Nuclear extracts underwent SDS-PAGE gel electrophoresis and were transferred onto PVDF membranes. Membranes were incubated overnight with HIF-1 alpha antibody (1/1500; kindly provided by the laboratory of Dr. Darren Richard, Laval University, QC, Canada), in 1% milk buffer solution (1 M Tris, 5 M NaCl). After washing (1 M Tris, 5 M NaCl, 0.1% Triton), the membranes were incubated with the secondary antibody (anti-IgG rabbit, 1/15 000; also provided by Dr. Richard) in a 1% milk buffer solution, for one hour. Antibody against β-actin (1/25 000 dilution, Novus Biologicals, Oakville, ON, Canada) was used as control. Protein bands were developed with an enhanced chemiluminescence substrate (PerkinElmer Inc, Waltham, MA, United States) and quantified using ImageJ 1.36b (http://rsbweb.nih.gov/ij/), with values normalised against β-actin expression.

### Statistical analysis

Data were analysed using GraphPad Prism v.5 for Windows (GraphPad Software; San Diego, CA, USA), and are presented as the mean ± SEM. Differences between the NNI, NH and H groups (males and females) were analysed using a two-way analysis of variance (ANOVA) with the factors hyperoxia exposure (p_H_), sex (p_S_), and their interaction (p_HxS_). In the assessment of DMOG treatment in NH and H groups, data was also analysed using a two-way ANOVA with the factors hyperoxia exposure (p_H_), DMOG treatment (p_DMOG_) and their interaction (p_HxDMOG_); males and females were analysed separately. To determine differences between individual groups, each two-way ANOVA was followed by a Bonferroni post-hoc test. To control for any litter effect, in cases where more than one animal per litter was assessed only the mean of those animals was used in the statistical analysis. In the assessment of the semi-quantitative measures (HIF-1α score) a Mann-Whitney test was performed. Statistical significance was accepted at the level of p < 0.05.

## Results

### Impact of neonatal hyperoxia

**Body and kidney weight.** There was no effect of hyperoxia exposure on body weight, kidney weight, or kidney weight to body weight ratio in pups at P5 or P10 of age; there was also no difference between males and females at either time point (Table 1).

**Table 1:** Average body weight, kidney weight, and kidney to body weight ratio in H, NH and NNI pups at P5 and P10, and in NaCl and DMOG treated NH and H animals at P5 and P10.

	Body Weight (g)		Kidney Weight (g)		Kidney to Body Weight Ratio (mg/g)	
***P5***	***Male***	***Female***	***Male***	***Female***	***Male***	***Female***
**NNI**	9.61±0.18	9.02±0.43	0.059±0.002	0.056±0.004	6.08±0.39	6.24±0.60
**NH**	10.39±0.34	9.90±0.29	0.064±0.007	0.069±0.003	6.15±0.55	6.92±0.16
**H**	9.64±0.85	9.22±0.77	0.050±0.008	0.057±0.008	5.16±0.88	6.16±0.48
***P10***	*Male*	*Female*	*Male*	*Female*	*Male*	*Female*
**NNI**	21.97±1.37	23.33±1.13	0.15±0.02	0.17±0.01	6.84±0.56	7.23±0.21
**NH**	21.33±0.81	22.17±2.16	0.15±0.01	0.17±0.03	7.21±0.23	7.41±0.48
**H**	23.60±1.96	21.60±1.23	0.16±0.02	0.14±0.02	6.62±0.54	6.42±0.48
***P5 DMOG***	***Male***	***Female***	***Male***	***Female***	***Male***	***Female***
**NH Control**	11.35±1.28	13.68±1.79	0.075±0.009	0.093±0.008	6.67±0.32	6.72±0.40
**NH+DMOG**	12.55±1.87	10.66±0.18	0.080±0.000	0.073±0.003	6.46±0.82	6.92±0.29
**H Control**	10.79±0.20	11.01±0.64	0.070±0.006	0.075±0.006	6.39±0.43	6.77±0.31
**H+DMOG**	10.17±0.46	9.99±0.47	0.070±0.004	0.070±0.006	6.67±0.19	7.06±0.19
***P10 DMOG***	***Male***	***Female***	***Male***	***Female***	***Male***	***Female***
**NH Control**	31.52±0.94	32.15±0.75	0.21±0.01	0.22±0.01	6.65±0.35	6.75±0.29
**NH+DMOG**	28.66±0.99*	29.03±1.00*	0.19±0.01	0.20±0.01	6.75±0.17	6.91±0.23
**H Control**	31.92±0.57	32.12±1.05	0.21±0.01	0.22±0.01	6.54±0.20	6.82±0.16
**H+DMOG**	29.55±0.23*	29.87±0.85*	0.20±0.01	0.21±0.01	6.67±0.21	7.08±0.18

Data shown as mean ± SEM. *p<0.01 DMOG versus Control.

**Figure 1:**
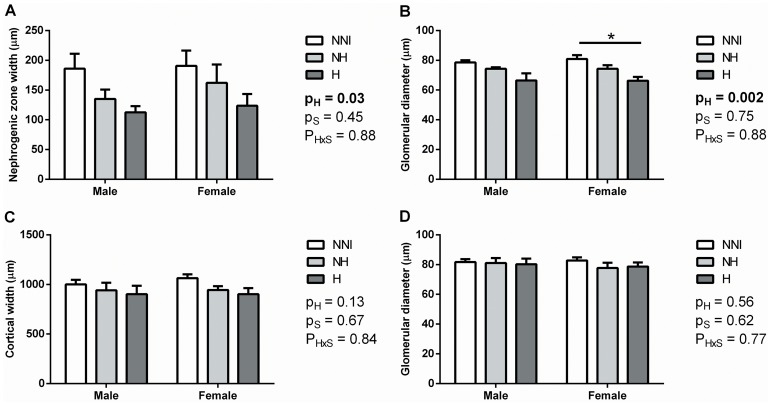
Nephrogenic zone width (A), glomerular diameter (B, D) and cortical width (C) in male and female animals from control (NNI, NH) and hyperoxia-exposed (H) groups at P5 (A-B) and P10 (C-D). There was a significant effect of hyperoxia exposure (p_H_) on nephrogenic zone width and glomerular diameter at P5 (A-B), but no effect at P10 (C-D). There was no effect of sex (p_S_) on any parameter. *p<0.05 according to Bonferroni post-hoc analysis.

**Renal morphology.** Hyperoxia exposure led to a significant reduction in nephrogenic zone width at P5 (Figure 1A); in males, nephrogenic zone width averaged 185.8±25.2 µm in the NNI control group, 135.1±15.8 µm in the NH group (a reduction of 27%), and 112.7±10.3 µm in the H group (a reduction of 39%). Similarly, there was a significant effect of hyperoxia exposure on glomerular diameter at P5 (Figure 1B). In animals at P10, however, there was no effect of hyperoxia exposure on the width of the renal cortex, or glomerular diameter (Figure 1C-D). There was no effect of sex on nephrogenic zone width, cortical width, or glomerular diameter in animals at P5 or P10.

**Figure 2:**
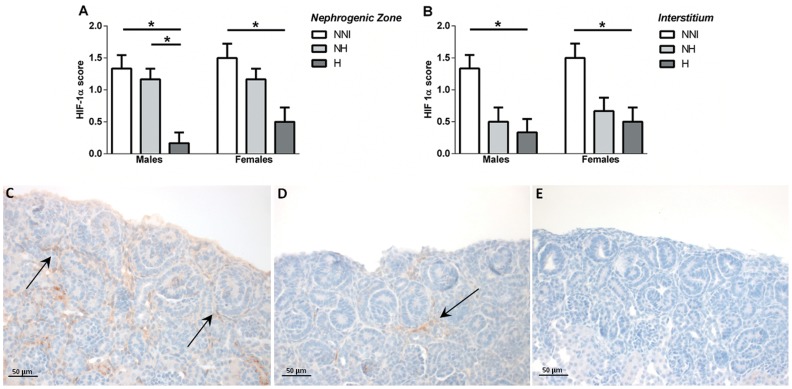
HIF-1α score (indicating the percentage of cells positive for HIF-1α expression) in the nephrogenic zone (A) and the cortical interstitium (B) in male and female animals from control (NNI, NH) and hyperoxia-exposed (H) groups at P5. *p<0.05 between groups as indicated. Representative photomicrographs of HIF-1α expression (indicated by brown staining; arrows) in the nephrogenic zone of NNI (C), NH (D) and H animals (E) at P5.

**HIF 1α expression.** As shown in Figure 2 (C-E), the expression of HIF-1α was localised to the nuclei of cells within the renal cortex of the developing kidney at P5. Within the nephrogenic zone (Figure 2A), and the cortical interstitium (Figure 2B), the number of cells positive for HIF-1α expression was significantly reduced in H animals compared to animals in the NNI control group.

**Figure 3:**
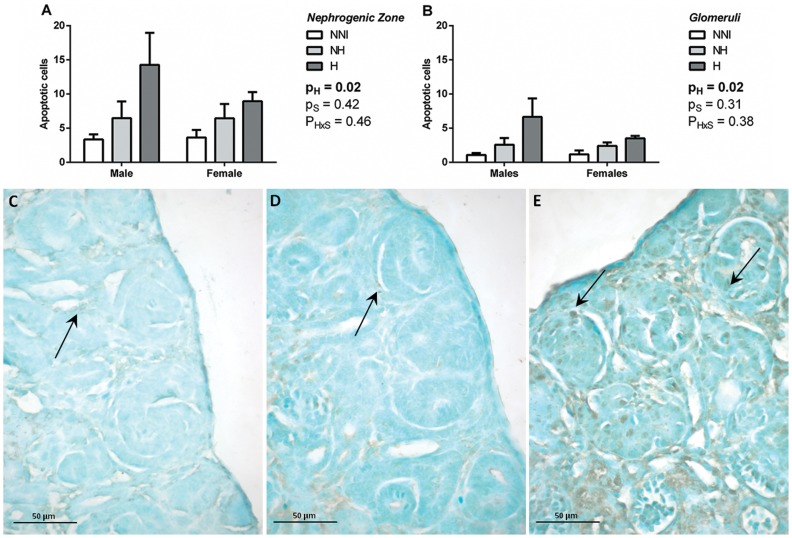
The number of apoptotic cells in the area of nephrogenic zone (A) and glomeruli (B) of male and female animals from control (NNI, NH) and hyperoxia-exposed (H) groups at P5. There was a significant effect of hyperoxia exposure (p_H_) on the number of apoptotic cells, but no effect of sex (p_S_). Representative photomicrographs of TUNEL staining of apoptotic cells (indicated by brown staining; arrows) in the nephrogenic zone of NNI (C), NH (D) and H animals (E) at P5.

**Apoptosis.** Overall, there was a significant effect of hyperoxia exposure on the number of apoptotic cells in the nephrogenic zone (Figure 3A), and within glomeruli of the nephrogenic zone (Figure 3B), in animals at P5.

### Impact of DMOG treatment

**Body and kidney weight.** At P5, there was no significant effect of DMOG treatment on body weight, kidney weight, or kidney weight to body weight ratio (Table 1). At P10, however, body weights were significantly reduced in male and female NH and H animals treated with DMOG compared to saline controls (Table 1). There was no difference between groups in kidney weight or kidney weight to body weight ratio at P10.

**Figure 4:**
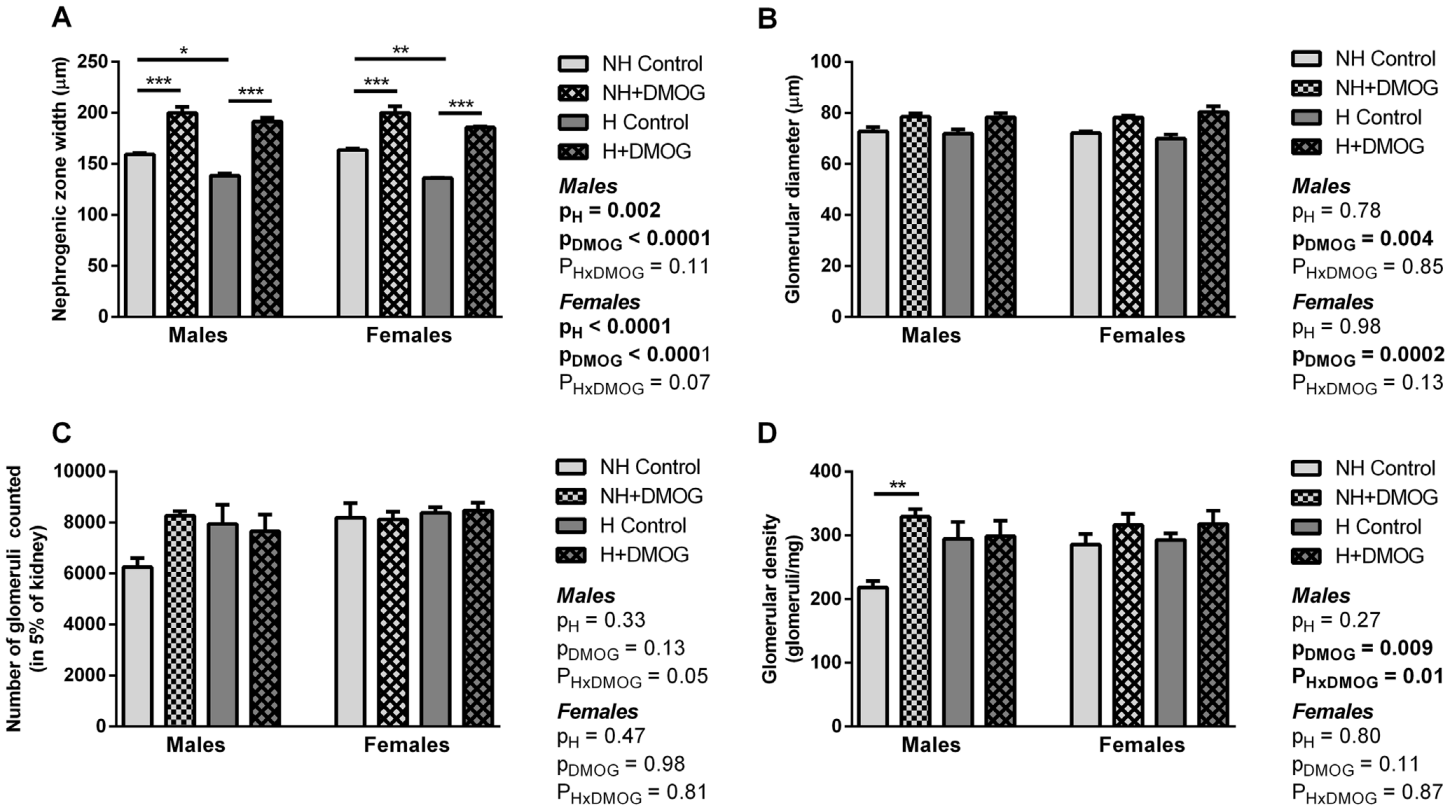
Nephrogenic zone width (A) and glomerular diameter (B) in NH and H males and females at P5 that were treated with DMOG, compared to saline-treated controls. There was a significant effect of hyperoxia exposure (pH) and DMOG treatment (pDMOG) on nephrogenic zone width in males and females (A). There was a significant effect of DMOG treatment (pDMOG), but no effect of hyperoxia (pH), on glomerular diameter in both males and females (B). The number of counted glomeruli in 5% of the kidney (C) and glomerular density (D) in in NH and H males and females at P5 that were treated with DMOG, compared to saline-treated controls. There was no significant difference in glomerular number between groups, but glomerular density was significantly increased in NH+DMOG males. *p<0.05, **p<0.01, ***p<0.001 according to Bonferroni post-hoc analysis.

**Renal morphology.** As shown in Figure 4A, both hyperoxia exposure and DMOG treatment had a significant effect on nephrogenic zone width in both males and females. A significant increase in nephrogenic zone width was evident in DMOG-treated animals; in H males, nephrogenic zone width averaged 138.5±2.2 µm in saline-treated controls, but was shown to increase 28% to 191.6±3.6 µm in the DMOG-treated animals (Figure 4A). DMOG treatment also had a significant effect on glomerular diameter at P5, with increased glomerular diameter evident in DMOG-treated male and female animals from both NH and H groups (Figure 4B).

**Glomular number and density.** At P10, there were no significant differences between groups in the number of glomeruli counted in 5% of each kidney (Figure 4C). Glomerular density, however, was significantly increased in NH males treated with DMOG compared to NH controls (Figure 4D).

**Figure 5:**
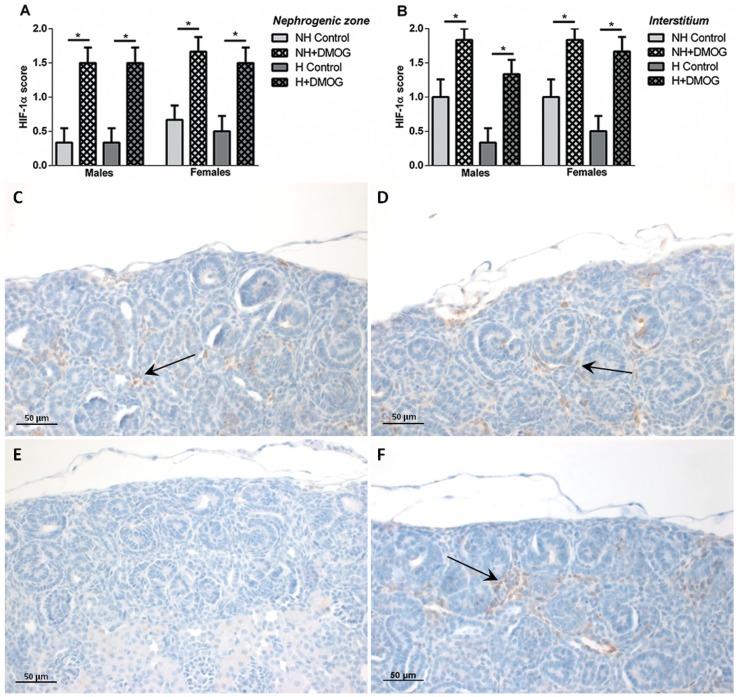
HIF-1α score (indicating the percentage of cells positive for HIF-1α expression) in the nephrogenic zone (A) and the cortical interstitium (B) in NH and H males and females at P5 that were treated with DMOG, compared to saline-treated controls. *p<0.05 between groups as indicated. Representative photomicrographs of HIF-1α expression (indicated by brown staining; arrows) in the nephrogenic zone of NH Control (C), NH+DMOG (D), H Control (E) and H+DMOG (F) animals at P5.

**HIF-1α expression.** Within the nephrogenic zone, and the cortical interstitium, the number of cells positive for HIF-1α expression was significantly increased in DMOG-treated animals compared to saline-treated controls, within both the NH and H groups (Figure 5); a similar pattern was observed in both males and females. In the Western blot analyses of nuclear extracts, two bands at approximately 116 kDa and 140 kDa were observed, which likely represent HIF-1a and ubiquitinated-HIF-1a respectively [32]. The ratio of intensity (normalized to β-actin) of HIF-1α (116 kDa) averaged 23.7% lower in male, but 1.3% higher in female NH+DMOG animals compared to NH Controls; H+DMOG animals had average levels 7.2% higher in males, and 3.2% higher in females compared to H Controls. Ubiquinated-HIF-1α (140 kDa) levels averaged 5.5% and 10.5% higher in NH+DMOG compared to NH Control groups in males and females respectively. Similarly, levels averaged 26.1% higher in H+DMOG males and 21.2% higher in H+DMOG females compared to H Controls. There was no statistically significant differences in results between any treatment groups, or between sexes.

**Apoptosis.** There was no significant effect of DMOG treatment on the number of apoptotic cells within the nephrogenic zone, or glomeruli of the nephrogenic zone, in either males or females.

## Discussion

The findings of this study demonstrate that transient hyperoxia exposure in the neonatal period results in impaired nephrogenesis, with a reduced nephrogenic zone width, reduced glomerular size, and increased apoptosis in the neonatal rat kidney. Importantly, HIF-1α expression in the renal cortex was significantly reduced following hyperoxia exposure. We have shown that inhibiting the oxygen-induced degradation of HIF-1α results in an attenuation of the adverse effects of hyperoxia on renal development, highlighting the potentially important role of HIF-1α signaling in renal development.

### Neonatal hyperoxia exposure impairs nephrogenesis

The extrauterine environment represents a significant stress for underdeveloped organ systems, such as the preterm neonatal kidney in which nephrogenesis is ongoing after birth. Nephrogenesis *in utero* normally occurs at very low oxygen concentrations; after birth, blood oxygen levels quickly rise which can result in oxidative stress of the newborn, particularly with the use of supplemental oxygen therapy [16], [17]. Although postnatal nephrogenesis normally occurs in term-born rat pups breathing room air, we propose that exposure to high oxygen levels after birth augments the level of stress imposed on the immature kidneys and consequently models the condition of the preterm newborn. In this regard, we have previously shown that 80% O_2_ exposure in the neonatal period results in vascular rarefaction, hypertension, and reduced nephron number in the adult rat [13], similar to clinical outcomes seen in children and adults that were born preterm [3]–[5]. In contrast, a recent study in a mouse model (mice exposed to 65% O_2_ from birth to postnatal day 7) showed no changes in nephron number [33]; the differences in findings between studies may relate to differences in the concentration of oxygen used, the postnatal days of exposure, and also susceptibilities of the animal model.

The 25% reduction in nephron number observed in adulthood in this model [13] may be the result of an accelerated loss of nephrons, and/or impaired nephrogenesis. In argument for the latter, the findings of this study demonstrate that neonatal hyperoxia exposure results in a significant reduction in nephrogenic zone width at P5 (following 48 hrs of hyperoxia exposure). These findings are possibly indicative of an early cessation of nephrogenesis, as suggested by the similar findings of a reduced nephrogenic zone width (in addition to other indicators of accelerated postnatal renal maturation) in human neonates following preterm birth [2]. An exhausted population of renal progenitor cells has been postulated to be the most likely cause for the cessation of nephrogenesis [34]. In this regard, high oxygen levels have previously been shown to reduce the population of progenitor cells in the lung [35], and in renal epithelial cell culture, cells exposed to hyperoxia exhibit early signs of differentiation [36]. In the current study, we also observed a significantly increased number of apoptotic cells in the nephrogenic zone of the hyperoxia-exposed kidneys. Cell death (via apoptosis or necrosis) is one of the most predominant pathological findings following hyperoxia exposure, and is particularly evident in the lung [37], [38]. In a hyperoxic environment, apoptosis occurs following the increased formation of reactive oxygen species (ROS) by the mitochondria [37]. It is possible that both the cessation of nephrogenesis in combination with increased apoptosis may have contributed to the reduced nephrogenic zone width.

Exposure to high oxygen levels is also known to impair vascular development in organs such as the lung and retina [20], [21] with microvascular rarefaction of skeletal muscle also observed in this model [13]. It is conceivable that development of the glomerular capillaries during ongoing nephrogenesis may also be impaired, thus resulting in the reduced glomerular size observed in the current study. Previous studies in a baboon model of preterm birth [39], and in human preterm neonates [2], have shown that morphologically abnormal glomeruli with enlarged Bowman’s space and scant glomerular capillarisation are commonly observed in the outer renal cortex of the kidney following preterm birth, which supports the suggestion that glomerular development may be impaired (and thus contribute to accelerated, or an increased susceptibility to nephron loss in adulthood). No similar morphological evidence was seen in the current study, however, and the cause of these abnormalities is yet to be determined.

Alternative explanations for the impaired renal development following hyperoxia exposure include maternal/neonatal stress and growth restriction; extrauterine growth restriction is a common finding in premature neonates, who have a reduced postnatal growth rate compared to that of normal fetal growth *in utero* [40]. In general, nephron endowment is directly correlated with body growth [41], with both intrauterine and extrauterine growth restriction associated with impaired renal growth and function in humans [42], [43]. Importantly, however, we found no difference in body or kidney weights between groups in this study, suggesting that growth restriction was not the cause of the impaired renal development. The NH group (alternated dams exposed to hyperoxia for 12/24 hrs, normoxic pups) was included in this study in order to control for all stressful interventions, including maternal exposure to high oxygen levels. The general trend in all analyses was that the NH group exhibited intermediate findings between the control NNI and the hyperoxia exposed H groups; as adults, we have previously shown that nephron number in the NH group was significantly reduced compared to controls [13]. This suggests that O_2_ breathing by the lactating dam and/or neonatal or maternal stress can also impact renal development in the pups, through mechanisms that remain to be determined. The two factors (O_2_ exposed dam and stress of interchange) cannot be separately assessed in this study, however we have previously shown that interchanging control (NNI) dams as the sole intervention did not impact offspring blood pressure or nephron number in adulthood [13] which is suggestive of a primary role of maternal O_2_ exposure.

In contrast to the findings of a reduced nephrogenic zone width and decreased glomerular size at P5, animals assessed at P10 (after the completion of nephrogenesis) did not exhibit a reduction in glomerular size, or a significant change in the width of the renal cortex. There was also no difference in the total number of glomeruli between NH and H groups. As the postnatal interventions were only imposed in the final stages of nephrogenesis, it is possible that the reduced nephrogenic zone width observed at P5 had a relatively minor impact on overall renal growth. Our previous finding of a reduced nephron number in adulthood [13] in this model may therefore be primarily explained by accelerated nephron loss rather than an observable impact on nephron endowment at the immediate completion of nephrogenesis. Potentially, the change in glomerular size may represent an incipient phase of compensatory renal hypertrophy following the initially suboptimal nephrogenesis. It is also to be noted that in this model, 4–6 pups per litter were sacrificed at P5, with the remainder continued until P10. This reduction in litter size may have had an impact on animal growth and renal development; future studies involving the assessment of whole litters at each time point would be required in order to investigate this factor.

### The role of HIF-1α in nephrogenesis

HIF-1α expression was shown to be markedly reduced following hyperoxia exposure in both the nephrogenic zone and cortical interstitium of H males and females, compared to normoxic controls. This result was certainly expected due to the known role of oxygen tension in the control of HIF-1α protein levels, whereby under normoxic and hyperoxic conditions HIF-1α is rapidly hydroxylated by prolyl hydroxylase domain (PHD) proteins which results in its ubiquitination [23], [24]; under hypoxic conditions, as occurs during renal development, HIF-1α is stabilised [44]. Even though logically expected, this is, to our knowledge, the first study to demonstrate that exposure to high oxygen concentrations *in vivo* markedly decreases HIF-1α expression in the kidney. The exact function of HIF-1α during nephrogenesis is largely unknown [45], but the localisation of expression to the nephrogenic zone suggest that it plays an important role. We observed nuclear expression of HIF-1α in cells located within the nephrogenic zone of the neonatal rat kidney, which corresponds with a previous study that demonstrated strong HIF-1α expression in the nuclei of ureteric bud epithelia, and in cells of the proximal portion of S-shaped bodies within the nephrogenic zone of developing human kidneys [46].

In order to determine what effects HIF signaling had on nephrogenesis in this model, we counteracted the oxygen-induced reduction in HIF-1α via systemic treatment with DMOG (a PHD inhibitor). In accordance with a previous study, we found (via immunohistochemical analysis) that systemic DMOG treatment resulted in a significant increase in HIF-1α expression in the kidney [47]; increased HIF-1α levels were observed in both NH control and H (hyperoxia-exposed) animals, within the nephrogenic zone and cortical interstitium of the developing kidney at P5. Although western blot analyses of HIF-1α protein expression did not show a statistically significant difference between groups (and was complicated by the presence of the two separate bands), the general pattern of expression was similar to that of the immunohistochemistry results. Importantly, the observed adverse effects of hyperoxia exposure on nephrogenesis were shown to be abrogated by the DMOG treatment (and consequent increase in HIF-1α expression) which highlights the role of HIF signaling during nephrogenesis. Both nephrogenic zone width and glomerular size were increased in the hyperoxia-exposed DMOG-treated animals to levels equivalent to the NNI normoxic controls. Interestingly, an increase in nephrogenic zone width and glomerular size also paralleled a significant increase in HIF-1α expression in the NH (normoxic) controls following DMOG treatment. This finding indicates that augmenting HIF-1α levels in the normoxic environment may be able to enhance ongoing postnatal nephrogenesis. Indeed, the increase in oxygen tension after birth has been previously postulated to trigger the cessation of nephrogenesis in rodents [48]; determining the exact role of HIF signaling in renal development is certainly an important area for future research.

In animals assessed at P10, DMOG treatment had no effect on the total number of glomeruli. As discussed above, given that DMOG treatment was only administered at the latter stages of nephrogenesis, it may be expected that the resultant improvements at P5 may not have ultimately resulted in an observable augmentation of nephron endowment. Furthermore, as animals were only administered the DMOG at P2 and P4, it is possible that the treatment protocol was not able to fully counteract the oxygen-induced reduction in HIF-1α expression for the full 7 days of hyperoxia exposure. Glomerular density, however, was significantly increased in DMOG-treated NH males compared to saline controls, but no significant difference was observed in females. In accordance with the positive effects on nephrogenesis observed at P5, this finding may be indicative that postnatal HIF stabilization increases nephron endowment in normoxic males; alternatively, given the relatively low numbers of glomeruli in that group (see Figure 4), the nephron density of NH males administered saline may have also been decreased by an unknown mechanism.

HIF-1α is known to be a major transcriptional regulator of genes involved in nephrogenesis, in particular vascular endothelial growth factor (VEGF) which is an essential factor in the development of the renal vasculature and tubules [49]–[52]. Given the importance of HIF-1α and VEGF signaling in vascular development [27], [46], it is possible that decreased VEGF expression as a result of the low HIF-1α levels may have contributed to the reduced glomerular size observed following hyperoxia exposure in the current study. Future studies involving ultrastructural analyses of the glomeruli and renal vasculature would be important in order to determine whether glomerulogenesis is adversely affected, as well as follow-up studies to determine whether this does result, as hypothesized, in accelerated nephron loss. HIF-1α also transcriptionally controls the expression of a multitude of genes involved in cell proliferation, differentiation, and apoptosis [27], [53] which supports an extended role for HIF in nephrogenesis besides its known function in vasculogenesis and angiogenesis. In the current study, however, DMOG treatment did not have a significant effect on the number of apoptotic cells in the developing kidney.

Throughout this study we focused on HIF-1α as it had previously been shown to be expressed in the nephrogenic zone of the developing kidney [46]; however, it is possible that another HIF isoform, HIF-2α, may also be involved in the observed improvements in glomerular size and nephrogenic zone width following DMOG treatment in the current study. In the developing kidney, HIF-2α expression is limited to interstitial cells of the medulla, podocytes, and endothelial cells [46]; the localisation of HIF-2α to the glomeruli suggests a possible role in the development and/or maintenance of glomerular capillaries. In other studies, HIF-2α has been shown (independently of HIF-1α) to be involved in the regulation of Oct4 (stem cell factor) expression [54], to regulate antioxidant gene expression [55], and it is also protective against ischaemia-reperfusion injury in the kidney [56] but overall its role in renal development remains uncertain [45]. Additionally, it should be noted that besides the prevention of oxygen-induced HIF degradation, PHD inhibitors such as the oxoglutarate analogue DMOG likely independently influence other pathways, including NFKB signaling [57]. In this regard, the body weight of DMOG-treated animals was significantly reduced at P10 suggestive of an adverse effect on growth (either via biochemical changes, or perhaps through peritoneal irritation following injection). Further studies are required in order to fully elucidate the role of the HIF isoforms (and/or PHD) in nephrogenesis and apoptosis.

## Conclusions

Overall, the findings of this study support the premise that neonates exposed prematurely to the oxygen-rich extrauterine environment are vulnerable to impaired renal development. We have shown that transient hyperoxia exposure, at a time when renal development was still ongoing, results in impaired nephrogenesis in the neonatal rat kidney. Although nephron number was not significantly reduced at the completion of nephrogenesis, the early indicators of maldevelopment suggest the potential for later nephron loss. Importantly, hyperoxia exposure significantly reduced HIF-1α expression. By using DMOG treatment to prevent the oxygen-induced reduction in HIF-1α we have shown that the adverse effects of hyperoxia exposure on nephrogenesis may be HIF-1α-mediated. Maintaining HIF-1α levels in the kidney may allow ongoing nephrogenesis to proceed and improve the long-term renal health of infants born preterm.
